# Commentary on “Interaction in Spoken Word Recognition Models”

**DOI:** 10.3389/fpsyg.2018.01568

**Published:** 2018-08-30

**Authors:** Dennis Norris, James M. McQueen, Anne Cutler

**Affiliations:** ^1^MRC Cognition and Brain Sciences Unit, University of Cambridge, Cambridge, United Kingdom; ^2^Donders Institute for Brain, Cognition and Behaviour, Radboud University, Nijmegen, Netherlands; ^3^Max Planck Institute for Psycholinguistics, Nijmegen, Netherlands; ^4^MARCS Institute, Western Sydney University, Penrith, NSW, Australia

**Keywords:** speech perception, computer simulation, feedback, cognitive science, word recognition

Magnuson et al. (2018: MMLSH), responding to Norris et al. (2016: NMC16), postulate that feedback of activation from words to pre-lexical representations is helpful in spoken-word recognition. Their argument (1) is flawed by being bound to a particular class of model, (2) misses the central point about parsimony in recognition models, and (3) ignores crucial data.

1. MMLSH describe simulations with the interactive-activation model TRACE (McClelland and Elman, 1986). Activation feedback is a key feature of TRACE: activation feeds back from word-form representations to influence the activation of pre-lexical phoneme representations. The simulations show that (for most though not all words) feedback improves word recognition when noise is added to the input. As we will argue, however, this demonstration has no bearing on the larger theoretical question of whether activation feedback is necessary, or even helpful, in speech recognition (Norris et al., 2000: NMC00; NMC16). The MMLSH simulations do not show that activation feedback necessarily improves word recognition because showing that it helps TRACE does not entail that it will help other models.

If the frequency of all words is assumed to be the same, then the best that any speech recognition system can do is compute the match between input features and lexical representations and select the best-matching word (more specifically, pick the word with the maximum likelihood). Since words differ in frequency, however, priors are available. The task is then to compute the posterior probability of the words as the product of the likelihood and prior (i.e., use Bayes' rule). This is how Shortlist B (Norris and McQueen, 2008: NM08) works. Shortlist B is feedforward and, by virtue of implementing Bayesian inference, performs optimally; its use of Bayes' rule guarantees that the best-matching word must be recognized.

Why then can TRACE benefit from feedback? The inescapable conclusion is that TRACE does not perform optimally, as just defined. This is not surprising. TRACE's internal currency is not probability, but activation. As one of the developers of TRACE explained (McClelland, 1991, 2013), interactive-activation models do not compute posterior probabilities. Instead, the decision about which word is present depends on a response threshold set on the output of the Luce choice rule. Reaching this threshold depends on differences among the activations of different candidate words. Crucially, because there is no internal noise, feedback has free rein to amplify these differences in arbitrary ways. These activation values therefore do not reflect the posterior probabilities of words. Contrary to MMLSH's claim, TRACE's behavior is thus neither optimal nor Bayesian. In an optimal system operating on noisy input without the Luce choice rule, feedback will amplify both signal and noise, and hence will achieve nothing.

Indeed, as MMLSH's simulations show, adding feedback to TRACE has little effect when there is no noise in the input. Rather, what feedback does is protect the model's speed and accuracy against the negative effects of increasing noise: feedback from word to phoneme nodes amplifies initial differences in phoneme-node activations and this in turn amplifies differences in word-node activations, counteracting the reductions in those differences that increasing noise has caused. This helps TRACE because its initial behavior is suboptimal, but says nothing about the need to include feedback in other models.

MMLSH's discussion about whether activation feedback causes “hallucinations” is also model-specific. Activation feedback does not cause listeners to hallucinate indiscriminately, but it does run the risk of creating hallucinations (NMC00, NMC16). Parameters in TRACE can be adjusted to avoid these negative effects, but, as McClelland et al. (2014) showed, it takes a very different kind of interactive-activation model to behave in a fully Bayesian way. A model built from the start on Bayesian principles would need no such parameter tweaking and would always behave optimally anyway.

(2) MMLSH argue that, on a count of nodes and connections, models with activation feedback are simpler than those without it. TRACE actually performs very badly in such a count because of massive reduplication of nodes over time slices (Norris, 1994); this is why MMSLH had to exclude many activated nodes to keep their simulations within bounds (p. 5). If number of parameters is the metric used, Bayesian models (because of their strong principles) need far fewer free parameters than interactive-activation models (7 as opposed to 16, comparing the Bayes-based Merge B with the activation-based Merge A; NM08). The divergent performance of different metrics only emphasizes the pointlessness of making claims about the relative complexity of different models in an informal and arbitrary manner; such comparisons should be formal (c.f. Vandekerckhove et al., 2015) and use fully-specified models, as in the Merge A/B case.

Also on parsimony, MMLSH misinterpret NMC00's: “Information flow from word processing to these earlier stages is not required by the logic of speech recognition and cannot replace the necessary flow of information from sounds to words. Thus it could only be included […] as an additional component” (NMC00, p. 299). MMLSH curiously read “not required by logic” as “illogical” (Is loving your spouse required by logic? Certainly not, but that does not make it illogical). An accurate reading of “not required by logic” is, of course, “not necessary”, and this is the central point about parsimony: additional components should only be added if it is strictly necessary to do so. MMSLH do not address this point.

(3) Crucial behavioral evidence is inconsistent with activation feedback (McQueen et al., 2009; Kingston et al., 2016). MMLSH fail to discuss this evidence. MMSLH note neuroscientific findings, but such evidence is inconclusive, as it could arise from other types of feedback (e.g., for learning or binding; NMC16). These other types of feedback are helpful, may indeed be necessary in speech recognition, and, in some cases, are supported by evidence (e.g., feedback for learning, Norris et al., 2003). Activation feedback is the only type with a function that is not self-evident and which is confuted by existing evidence.

Theoretical arguments and the available empirical data thus indicate that activation feedback is not necessary in on-line speech recognition. Indeed, activation feedback is unable to improve the already optimal performance of any Bayesian feedforward model.

## Author contributions

All authors listed have made a substantial, direct and intellectual contribution to the work, and approved it for publication.

### Conflict of interest statement

The authors declare that the research was conducted in the absence of any commercial or financial relationships that could be construed as a potential conflict of interest.

## References

[B1] Kingston J. Levy J. Rysling A. Staub A. (2016). Eye movement evidence for an immediate Ganong effect. J. Exp. Psychol. Hum. Perc. Perf. 42, 1969–1988. 10.1037/xhp0000269 27736119

[B2] Magnuson J. S. Mirman D. Luthra S. Strauss T. Harris H. D. (2018). Interaction in spoken word recognition models: feedback helps. Front. Psychol. 9:369. 10.3389/fpsyg.2018.00369 29666593 PMC5891609

[B3] McClelland J. L. (1991). Stochastic interactive processes and the effect of context on perception. Cogn. Psychol. 23, 1–44. 10.1016/0010-0285(91)90002-6 2001614

[B4] McClelland J. L. (2013). Integrating probabilistic models of perception and interactive neural networks: a historical and tutorial review. Front. Psychol. 4:503. 10.3389/fpsyg.2013.00503 23970868 PMC3747375

[B5] McClelland J. L. Elman J. L. (1986). The TRACE model of speech perception. Cogn. Psychol. 10, 1–86. 10.1016/0010-0285(86)90015-0 3753912

[B6] McClelland J. L. Mirman D. Bolger D. J. Khaitan P. (2014). Interactive activation and mutual constraint satisfaction in perception and cognition. Cogn. Sci. 38, 1139–1189. 10.1111/cogs.12146 25098813

[B7] McQueen J. M. Jesse A. Norris D. (2009). No lexical–prelexical feedback during speech perception or: is it time to stop playing those Christmas tapes? J. Mem. Lang. 61, 1–18. 10.1016/j.jml.2009.03.002

[B8] Norris D. (1994). Shortlist: a connectionist model of continuous speech recognition. Cognition 52, 189–234. 10.1016/0010-0277(94)90043-4

[B9] Norris D. McQueen J. M. (2008). Shortlist B: a Bayesian model of continuous speech recognition. Psychol. Rev. 115, 357–395. 10.1037/0033-295X.115.2.357 18426294

[B10] Norris D. McQueen J. M. Cutler A. (2000). Merging information in speech recognition: feedback is never necessary. Behav. Brain Sci. 23, 299–325. 10.1017/S0140525X00003241 11301575

[B11] Norris D. McQueen J. M. Cutler A. (2003). Perceptual learning in speech. Cogn. Psychol. 47, 204–238. 10.1016/S0010-0285(03)00006-9 12948518

[B12] Norris D. McQueen J. M. Cutler A. (2016). Prediction, Bayesian inference and feedback in speech recognition. Lang. Cogn. Neurosci. 31, 4–18. 10.1080/23273798.2015.1081703 26740960 PMC4685608

[B13] Vandekerckhove J. Matzke D. Wagenmakers E. J. (2015). Model comparison and the principle of parsimony in The Oxford Handbook of Computational and Mathematical Psychology, eds Busemeyer J. R. Wang Z. Townsend J. T. Eidels A. (Oxford: Oxford University Press), 300–319.

